# Psychometric properties of the Spanish version of the Weight Self-Stigma Questionnaire (S-WSSQ) in a sample of participants with obesity seeking weight loss treatment

**DOI:** 10.1007/s40519-022-01511-6

**Published:** 2022-11-25

**Authors:** Alejandro Magallares, Pilar Benito de Valle, José Antonio Irles, Patricia Recio, Ignacio Jáuregui-Lobera

**Affiliations:** 1 School of Psychology, Social Psychology Department, Spanish Open University (UNED), Madrid, Spain; 2 Clinical Nutrition Unit, Hospital de Valme, Seville, Spain; 3 School of Psychology, Methodology Department, Spanish Open University (UNED), Madrid, Spain; 4 School of Experimental Sciences, Nutrition and Bromatology, Universidad Pablo de Olavide, Seville, Spain; 5 Departamento de Psicología Social y de las Organizaciones, Facultad de Psicología UNED, C/ Juan del Rosal, 10, 28040 Madrid, Spain

**Keywords:** Affect, Antifat attitudes, Life satisfaction, Obesity, Overweight, Self-esteem, Weight stigma, Weight Self-Stigma Questionnaire

## Abstract

**Purpose:**

Weight self-stigma may be defined as a self-devaluation due to one’s identification with the group of people with obesity. The Weight Self-Stigma Questionnaire (WSSQ) is an instrument specifically designed to measure weight self-stigma in populations with overweight or obesity. The objective of this study was to adapt the WSSQ to the Spanish population (S-WSSQ) following the guidelines for cross-cultural adaptations.

**Methods:**

The sample comprised 165 participants with obesity seeking weight loss treatment (65% women) at the “*Hospital de Valme*” (Seville, Spain). Scales to measure life satisfaction, self-esteem, positive and negative affect, and antifat attitudes were used to analyze the convergent and divergent validity of the S-WSSQ.

**Results:**

A confirmatory factor analysis showed adequate values of the goodness of fit indexes of a two-factor model (*χ*^2^/df = 2.01 CFI = 0.92, IFI = 0.92, SRMR = 0.08, RMSEA = 0.078), replicating the structure found by the original authors. Cronbach’s alphas of the two factors were 0.76 (self-devaluation) and 0.77 (fear of enacted stigma). Composite Reliability values were 0.72 (self-devaluation) and 0.76 (fear of enacted stigma). Self-devaluation and fear of enacted stigma were negatively related to self-esteem, and positive affect, and positively related to negative affect and antifat attitudes. Finally, life satisfaction was negatively correlated to fear of enacted stigma.

**Conclusions:**

Based on these results, it is concluded that the S-WSSQ has good psychometric properties and might be used by the Spanish-speaking scientific community to measure weight self-stigma.

**Level of evidence:**

Level V, descriptive study.

## Introduction

The prevalence of obesity and overweight has increased worldwide in the last few years [1]. Surprisingly, many studies find that weight stigmatization has risen as well during the last decades [2]. Indeed, research shows that obesity may be considered a social stigma [3], and that individuals with obesity have to face discrimination in many social areas [4], like at the workplace [5], in healthcare settings [6], in the schooling context [7], and at daily activities [8]. This widespread weight-related stigmatization usually leads to the development of weight self-stigma because people with obesity usually encounter weight stigma on a daily basis and are frequently aware of stigma directed at others who have a similar weight [9]. Previous studies have found that weight self-stigma, which may be defined as a self-devaluation due to one’s identification with the group of people with obesity [10], increases the risk of being socially rejected [8], plays a fundamental role in the non-adherence to weight loss treatments [11], and is also associated with poor health, both physical and psychological [12].

The Weight Self-Stigma Questionnaire (WSSQ) is an instrument specifically designed to measure weight self-stigma in populations with overweight or obesity that was originally developed in the United States [13]. The WSSQ has been validated so far in German [14], Chinese [15], Turkish [16], French [17], Italian [18], and Malaysian [19] populations. The original version of the WSSQ [13] showed a two-factor model, corresponding to self-devaluation (negative thoughts and emotions about being obese) and fear of enacted stigma (involves the perception of being discriminated, as well as the identification to a stigmatized group), and it had adequate reliability (with Cronbach’s alphas = 0.81 and 0.87, respectively). The French version [17] revealed excellent reliability for both WSSQ subscales (Cronbach’s alphas = 0.90 and 0.92), and similar results were also described in the Malaysian adaptation (Cronbach’s alphas = 0.89 and 0.91) [19]. In addition, a good reliability was found in the German (Cronbach’s alphas = 0.81 and 0.83) [14], Chinese (Cronbach’s alphas = 0.89 and 0.88) [15], and Italian (Cronbach’s alphas = 0.81 and 0.82) versions of the WSSQ for both factors. Finally, the Turkish translation of the WSSQ showed acceptable reliability for both components (Cronbach’s alphas = 0.74 and 0.81) [16].

### Research overview

To the best of our knowledge, there is no Spanish adaptation of the WSSQ to date. Considering that Spanish ranks second among languages in worldwide prevalence with more than 480 million speakers [20], we believe that a Spanish version of this questionnaire would facilitate examining the cultural universality of the weight self-stigma construct. Therefore, the main objective of the present study was to adapt to the Spanish context of the WSSQ following the guidelines for translation and cross-cultural adaptations [21].

Furthermore, to test the factor structure of the WSSQ, a confirmatory factor analysis (CFA) was conducted. According to the reviewed literature, we expected to find a two-factor structure that will mirror the original version of the WSSQ [13] and previous cross-cultural adaptations of the questionnaire [14–19]. In addition, to analyze the reliability of the S-WSSQ, Cronbach’s alphas and the Composite Reliability (CR) of the factors of the questionnaire were calculated.

Moreover, it was decided to study the convergent and divergent validity of the S-WSSQ. Significant relations between the WSSQ subscales and measures of disordered eating, general experiential avoidance, quality of life, psychological distress, and attitudes toward people with obesity were found in the original version [13]. The German adaptation demonstrated relations between the components of the WSSQ and measures of depression, quality of life, psychological distress, dissociative symptoms, and weight-related shame and guilt [14]. The Chinese version described that the factors of the WSSQ were correlated with body image [15]. The Turkish adaptation showed significant correlations with the two subscales of the WSSQ and depression, anxiety, eating behaviours disorders, self-esteem, and quality of life [16]. The French version found that the components of the WSSQ were correlated with measures of self-esteem, physical appearance anxiety, depression, fear of negative appearance evaluation, and eating-related pathology [17]. The Italian version of the WSSQ showed that both subscales were associated with body uneasiness and depression [18], and the Malaysian version reported a correlation between the subscales of the questionnaire and psychological distress [19].

In our study, it was decided to analyze the relationships that may exist between the S-WSSQ and life satisfaction, self-esteem, positive and negative affect, and antifat attitudes, because previous research has shown that weight self-stigma is related to less life satisfaction [22], predicts lower self-esteem [16, 17], leads to less positive/more negative affect [23], and is positively related to antifat attitudes [13]. Finally, gender differences in the S-WSSQ were also assessed, and for the rest of the variables of the study, since higher levels of weight self-stigma among women had been previously described [13].

## Methods

### Data collection

The recruitment procedure started when the therapists of the Clinical Nutrition Unit (CNU) of the “*Hospital de Valme*” (Seville, Spain) invited patients to participate in this study. Initially, 189 patients were asked to participate and only 19 of them refused to enroll in the study (11.7%). This participation rate (88.3%) may be considered good [24]. Five participants were eliminated from the final sample because of their weight (BMI ≤ 30) to the make sample more homogenous. These 165 adult participants had Spanish as their mother tongue and none of them withdrew from the study. All participants received weight management treatment as outpatients. These patients volunteered to take part in the study and none of them received any kind of reward after fulfilling the task. Data were collected anonymously and analyzed in an aggregated way.

With respect to the inclusion/exclusion criteria, all patients who were attending the CNU regularly (at least 80% of their medical appointments) and with good adherence, which may be defined as the extent to which a person’s behavior corresponds with the agreed recommendations from a health care provider, were invited to participate [25]. In addition to these premises, it should be considered that when a patient does not come regularly for consultation (being cited) and does not adhere adequately to treatment, he/she does not seem to be the most suitable candidate to participate in a study such as the present one. Based on these variables related to good adherence, therapists considered the pertinence (or not) to enroll candidates in this study. Considering age, all possible participants under legal age were excluded. Patients with other diagnostics (for example eating disorders, dysphagia, or malabsorption syndromes) were excluded as well as those who were not able to follow the ambulatory treatment due to medical complications or mental disorders which caused difficulties to go on a diet.

After having obtained the CNU Headmaster’s permission and the patients’ written informed consent, participants fulfilled the questionnaires individually and without time limits. The procedure was supervised by a nutritionist, instructing the participants about how to complete the questionnaires until they were absolutely sure about their fully understanding of the instructions. The participants developed their task in a suitable setting (adequate therapeutic context considering a spacious room and good conditions of light, noise, etc.). Anthropometric measurements (weight, height) were taken by nurses and dieticians of the CNU with enough experience in clinical research support.

### Sample

The final sample comprised 165 participants from the CNU of the “*Hospital de Valme*” (Seville, Spain) with a mean age of 46.69 years (SD = 13.01; Median = 46; Skewness = 0.08; Kurtosis = − 0.69) and a range between 20 and 73 years. The mean BMI was 43.18 kg/m^2^ (SD = 8.05; Median = 41.67; Skewness = 0.59; Kurtosis = − 0.05) with a range between 30.02 and 66.74 kg/m^2^.

108 were women and 57 were men. In women, the mean age was 45.79 (SD = 12.92; Median = 44.50; Skewness = 0.18; Kurtosis = − 0.67) with a range between 20 and 73, and the mean BMI was 43.07 (SD = 7.81; Median = 42.09; Skewness = 0.53; Kurtosis = 0.01) with a range between 30.02 and 66.74 kg/m^2^, while in men the mean age was 48.40 (SD = 13.12; Median = 48; Skewness = − 0.12; Kurtosis = − 0.55) with a range between 20 and 73 and the mean BMI was 43.41 (SD = 8.57; Median = 41.67; Skewness = 0.67; Kurtosis = − 0.13) with a range between 30.67 and 64.70 kg/m^2^.

### Adaptation procedure

The WSSQ was adapted to Spanish following the usual translation/back-translation methodology as well as the norms of the International Test Commission [21]. The first Spanish translation of the original questionnaire was performed by one of the authors of the current study. This Spanish translation was independently reviewed by another researcher, who worked with the first translator to reach an agreed-upon translation of the items, especially those which posed the most difficulty from the semantic and/or grammatical standpoint. Both conceptual and item equivalence were assessed through a literature review. Then, a bilingual English translator back-translated to English the agreed Spanish translation, with no knowledge of the original questionnaire in English to preserve the reliability of the back-translation. Items can be seen in Table 1.

**Table 1:** Items of the WSSQ: English and Spanish version

	WSSQ	S-WSSQ
	**Self-devaluation**	**Auto-devaluación**
1	I’ll always go back to being overweight	Siempre volveré a tener sobrepeso
2	I caused my weight problems	Soy el causante de mis problemas de peso
3	I feel guilty because of my weight problems	Me siento culpable por mis problemas de peso
4	I became overweight because I’m a weak person	Me convertí en una persona con sobrepeso porque soy débil
5	I would never have any problems with weight if I were stronger	Nunca habría tenido problemas de peso si fuera más fuerte
6	I don’t have enough self-control to maintain a healthy weight	No tengo suficiente autocontrol como para mantener un peso saludable
	**Fear of enacted stigma**	**Miedo al estigma**
7	I feel insecure about others’ opinions of me	Me siento inseguro sobre las opiniones de otros sobre mí
8	People discriminate against me because I’ve had weight problems	La gente me discrimina porque he tenido problemas de peso
9	It’s difficult for people who haven’t had weight problems to relate to me	Es difícil para personas que no hayan tenido problemas de peso relacionarse conmigo
10	Others will think I lack self-control because of my weight problems	Los demás creerán que me falta autocontrol por mis problemas de peso
11	People think that I am to blame for my weight problems	La gente cree que yo soy el culpable de mis problemas de peso
12	Others are ashamed to be around me because of my weight	Los demás están avergonzados de estar cerca de mí por mi peso

### Measures

To measure weight self-stigma, the Spanish translation of the WSSQ [13] was used. The WSSQ contains 12 items and it is divided into two major factors. The first group includes questions related to self-devaluation and the second one is related to fear of enacted stigma. Examples of items can be seen in Table 1. The items were rated on a 5-point Likert scale from 1 (“*strongly disagree*”) to 5 (“*strongly agree*”). Coefficient alphas of the original WSSQ were 0.81 (self-devaluation) and 0.86 (fear of enacted stigma) [13]. Self-devaluation and fear of enacted stigma scores were obtained by averaging the items of self-devaluation or fear of enacted stigma factors, respectively. Higher scores on self-devaluation reflect more negative thoughts and emotions about being obese. Higher scores on fear of enacted stigma reflect a greater perception of being discriminated.

To measure life satisfaction, the Life Satisfaction Scale (LSS) (English version [26]; Spanish version [27]) was used. This scale has 5 items and it had good reliability in our sample (Cronbach’s alpha = 0.82). A 5-point Likert scale (from 1-*strongly disagree*- to 5-*strongly agree*-) was used. An example of items would be “The conditions of my life are excellent”. The final score was obtained by averaging the scores on the 5 items of the scale. Higher scores on LSS reflect greater satisfaction with one’s life as a whole.

To measure self-esteem, Rosenberg’s Self-Esteem Scale (RSES) (English version [28]; Spanish version [29]) was used. This scale is a 10-item measure with a good reliability in our sample (Cronbach’s alpha = 0.84). A 5-point Likert scale (from 1-*strongly disagree*- to 5-*strongly agree*-) was used. An example of items would be “I feel that I have a number of good qualities”. The final score was computed by averaging the scores on the 10 items of the scale. Higher scores on RSES reflect a greater person's overall subjective emotional evaluation of his or her own worth.

To measure positive and negative affect, the Positive Affect and Negative Affect Scale (PANAS) (English version [30]; Spanish version [31]) was used. The PANAS is a 20-item instrument that evaluates positive (10 items) and negative affect (10 items). It is answered by means of a 5-point Likert scale (from 1-*never*- to 5-*always*-). “Enthusiastic” or “Nervous” would be examples of positive and negative adjectives, respectively. Positive and negative affect final scores were obtained by averaging the items of positive or negative affect scales, respectively. In our sample, Cronbach’s alpha was 0.83 for the positive affect subscale and 0.86 for the negative affect subscale. Higher scores on positive affect reflect greater positive feelings. Higher scores on negative affect reflect greater negative feelings.

To measure antifat attitudes, the Antifat Attitudes Questionnaire (AFA) (English version [32]; Spanish version [33]) was used. The AFA evaluates attitudes toward individuals with overweight and obesity. AFA (Cronbach’s alpha in our sample was 0.69) consists of 7 items scored on a 5-point Likert scale ranging from *strongly disagree* (1) to *strongly agree* (5). An example of items would be “Fat people make me somewhat uncomfortable”. A final score was obtained by averaging the 7 items of the scale. Higher scores on the AFA scale reflect greater dislike toward people with obesity.

BMI was calculated as the relationship between weight (Kg) and height squared (m^2^) (taken by nurses and dieticians of the CNU). Weight and height were taken individually, with participants in standing position, barefoot, and in light garments. A stadiometer (Atlántida S13; Básculas y Balanzas Añó-Sayol, Barcelona, Spain) was used. Finally, participants’ age, and gender were collected.

### Data analysis

The SPSS 25.0 statistics software and AMOS 25.0 program were used to conduct the following analyses. A series of prior calculations were obtained. First, the skewness and kurtosis of the variables were assessed to test whether the data matched a normal distribution. Values between − 2 and + 2 for both properties were considered proof of a normal univariate distribution [34]. Secondly, the sample adequacy of the variables in our study was analyzed. To assess the appropriateness of using factor analysis on the data set, the usual approach is to calculate Bartlett’s sphericity test and the Kaiser–Mayer–Olikin measure of sampling adequacy (KMO). According to experts, in the case of Bartlett’s sphericity test, when the null hypothesis is accepted (*p* > 0.05) it means that the variables are not intercorrelated and therefore it is not recommended to perform a factor analysis [35]. For the KMO measure, values between 0.70 and 0.80 may be considered good [35]. Data were screened for multivariate outliers with none found.

Once prior checks were completed, a CFA was conducted using the Maximum Likelihood (ML) estimation method. We chose to use a confirmatory strategy as we had a plausible hypothesis about the structure of the model [36]. To evaluate the overall model fit, several goodness-of-fit indices were calculated. These indices were the Comparative Fit Index (CFI), Incremental Fit Index (IFI), Standardized Root Mean Residual (SRMR) and Root Mean Square Error of Approximation (RMSEA). A CFI value greater than 0.90 is considered a psychometrically acceptable fit to the data [37]. IFI values above 0.90 show a reasonably good fitting model. SRMR and RMSEA are other quantitative indicators that describe how well the model fits the observed data. As a rule of thumb, a value of 0.08 or less indicates an acceptable model fit [37].

To assess the reliability of the S-WSSQ, the Cronbach’s alphas of the factors of the questionnaire were calculated. Reliability indexes ≥ 0.70, 0.80, or 0.90 are usually considered acceptable, good, or excellent, respectively [38]. In addition, the Composite Reliability (CR) was also calculated. According to experts, CR is a better reliability measure than Cronbach’s alpha in structural equation modeling, because it is based on the loadings rather than the correlations observed between the observed variables [39].

To analyze the convergent validity of the S-WSSQ, the relationships between weight self-stigma, negative affect, and antifat attitudes were studied by means of Pearson’s correlations. To study the divergent validity of the questionnaire, the association between weight self-stigma, life satisfaction, self-esteem, and positive affect was analyzed with the previous procedure. Finally, Student’s *t* tests were performed to explore gender differences in S-WSSQ, life satisfaction, self-esteem, positive and negative affect, antifat attitudes, BMI, and age. Cohen’s *d*s were also estimated to determine the effect size. According to experts, *d*s ≥ 0.4 are considered a moderate effect size [40].

## Results

According to the analyses performed (see Table 2), the 12 items of the S-WSSQ matched a normal distribution. Bartlett's test was 659.63 (66 *df*) with *p* < 0.001, and the KMO value was 0.76, which are both largely acceptable.

**Table 2:** Means, median, standard deviations (SD), skewness, and kurtosis of the items of the S-WSSQ

Items	Mean	Median	SD	Skewness	Kurtosis
1	2.68	3	1.58	.23	− 1.50
2	4.15	5	1.27	− 1.33	.52
3	3.98	5	1.38	− 1.13	− .05
4	3.02	3	1.68	− .02	− 1.67
5	3.00	3	1.68	.01	− 1.65
6	3.58	4	1.53	− .61	− 1.11
7	2.74	2	1.68	.27	− 1.62
8	2.25	1	1.59	.78	− .1.05
9	1.73	1	1.30	1.61	1.18
10	3.05	3	1.62	− .08	− 1.57
11	3.62	4	1.57	− .71	− 1.07
12	1.10	1	1.24	1.62	1.35

**Fig. 1:**
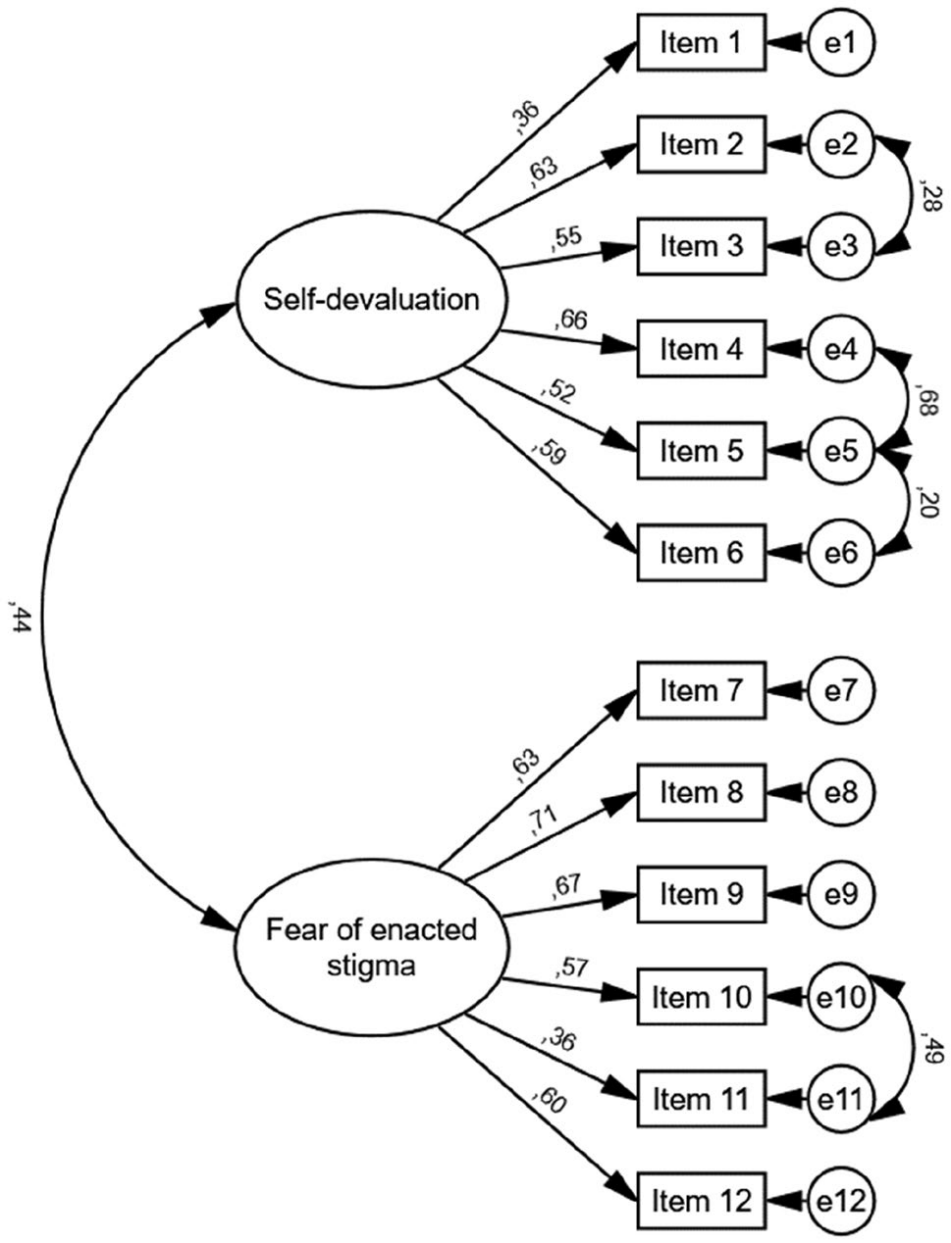
Confirmatory factor analysis. The values of the arrows are the standardized regression coefficients

Using CFA, we tested the two-factor solution that the original authors that developed the WSSQ originally found [13], which had 6 items per factor. The initial model did not fit the data (*χ*^2^/*df* = 3.56, CFI = 0.78, IFI = 0.78, SMRM = 0.098, RMSEA = 0.126). Thus, several model re-specifications were conducted on the initial model based on the modification indexes, using a sequential procedure. In the final model, errors of items 2–3, 4–5, 5–6, and 10–11 were correlated. Such correlated errors can occur due to specific item content [41]. Figure 1 shows the final CFA path diagram. The standardized values of regression coefficients were adequate, as the factor loadings were high and significant. Global goodness-of-fit indices showed a good model fit, as the relative chi-square value (chi-square value divided by the degrees of freedom) was 2.01, CFI was 0.92, IFI was 0.92, SRMR was 0.08 and RMSEA was 0.078 [37].

Once the structure had been established via the CFA, Cronbach’s alphas of the two factors were estimated. It was found a Cronbach’s alpha of 0.76 for self-devaluation and 0.77 for fear of enacted stigma. By sex, the internal consistency for self-devaluation was 0.77 for women and 0.74 for men, while for fear of enacted stigma was 0.76 for men and 79 for women. These values may be considered adequate [38]. In addition, CR values were 0.72 (self-devaluation) and 0.76 (fear of enacted stigma). Given that the threshold of acceptability for CR is 0.70, these results were also satisfactory [39].

Regarding the divergent validity of the S-WSSQ, negative and significant correlations for the two factors of the questionnaire and self-esteem and positive affect were found (see Table 3). In addition, life satisfaction was negatively related to fear of enacted of stigma. Regarding the convergent validity of the questionnaire, positive and significant correlations between self-devaluation, fear of enacted stigma, negative affect, and antifat attitudes were found. Finally, a positive and significant correlation between the two factors of the S-WSSQ (*r* = 0.44, *p* < 0.001) was found. Descriptives of all variables are shown in Table 4.

**Table 3:** Correlations between the S-WSSQ’s factors and the rest of the variables of the study

Variables	Self-devaluation	Fear of enacted stigma
Self-esteem	− .39***	− .63***
Life satisfaction	− .11	− .32***
Positive affect	− .29***	− .31***
Negative affect	.35***	.43***
Antifat attitudes	.27***	.29***

***p < .001

**Table 4:** Means, median, standard deviations (SD), skewness, and kurtosis of the variables of the study

	Mean	Median	*SD*	Skewness	Kurtosis
Self-devaluation	3.40	3.33	1.03	− .29	− .68
Fear of enacted stigma	2.51	2.33	1.03	.60	− .33
Self-esteem	3.81	4.10	.87	− .85	.03
Life satisfaction	2.96	3.00	1.08	− .03	− .88
Positive affect	3.42	3.50	.91	− .34	− .58
Negative affect	3.18	3.20	1.01	− .07	− .88
Antifat attitudes	1.52	1.28	.63	1.84	3.83

Finally, as it is shown in Table 5, women scored higher on both factors of the S-WSSQ but only the difference in self-devaluation was significant (*t* = 2.41, *p* = 0.015). Furthermore, gender differences were found in negative affect (*t* = 2.76, *p* = 0.006). Both differences may be considered as moderate according to Cohen’s *d*s found (− 0.40 and − 0.45, respectively) [40].

**Table 5:** Gender differences in the variables of the study

Variables	Men (*n* = 57)	Women (*n* = 108)	*t*	*d*
Self-devaluation	3.13 (1.01)	3.54 (1.01)	− 2.47*	− .40
Fear of enacted stigma	2.33 (1.07)	2.61 (1.01)	− 1.64	− .27
Self-esteem	3.94 (.83)	3.75 (.88)	1.41	.22
Life satisfaction	2.98 (1.17)	2.94 (1.03)	.23	.03
Positive affect	3.46 (.89)	3.39 (.83)	.45	.08
Negative affect	2.90 (.88)	3.33 (1.02)	− 2.76**	− .45
Antifat attitudes	1.49 (.56)	1.53 (.67)	− .49	− .06
BMI	43.40 (8.57)	43.07 (7.81)	.25	.04
Age	48.40 (13.12)	45.79 (12.92)	1.22	.20

*p < .05; **p < .01

## Discussion

According to the above-mentioned results, the main goal of this research, adapting the WSSQ to the Spanish population, was fulfilled. First, the WSSQ was successfully translated into Spanish [21]. Previous studies have assessed weight self-stigma with the WSSQ in the United States [13], Germany [14], China [15], Turkey [16], France [17], Italy [18], and Malaysia [19]. Our study adds to the effort made by other researchers to adapt the WSSQ to different cultural contexts.

Second, evidence of construct validity was obtained by means of confirmatory factor analysis. The CFA supported a two-factor model, which corresponded to the two dimensions originally proposed of self-devaluation and fear of enacted stigma [13]. The same two-factor solution has been found in all the translated versions of the questionnaire [14–19]. We believe that our research joins the work made by other colleagues to confirm the generalizability of the WSSQ factor structure.

Third, the reliability of the factors was appropriate given the Cronbach’s alphas [38] and CR values found [39]. Cronbach’s alphas obtained in our Spanish sample were very similar to the reliability found in the Turkish translation of this questionnaire [16]. However, they were slightly lower than the ones obtained by the original authors [13] and the previously mentioned versions of the WSSQ [14, 15, 17–19].

Fourth, evidence of convergent and divergent validity was obtained. It was found that self-esteem [16, 17], positive and negative affect [24], and antifat attitudes [13] were linked to self-devaluation and fear of enacted stigma. In addition, life satisfaction was negatively related to fear of enacted stigma [22]. Moreover, it was found that self-devaluation and fear of enacted stigma were positively related to each other, similarly to the description of the original authors [13]. Similar correlations between the factors of the WSSQ have been found in all the translated versions of the questionnaire [14–19].

Finally, it was found that women scored higher in the self-devaluation factor compared to men with a moderate effect size. The authors that developed the questionnaire also found that females experienced higher levels of self-devaluation and fear of enacted stigma [13]. This was an expected finding because women with obesity suffer more discrimination compared to men [42]. This result may explain why patients under treatment for obesity are oftentimes much more likely to be female than male [43].

### Practical implications

The authors that developed the WSSQ were particularly interested in an instrument that would be useful for studies of interventions targeting weight self-stigma [13]. According to these authors, the WSSQ allows researchers to measure the impact of interventions designed to alleviate weight self-stigma. For this reason, we believe that the S-WSSQ would be used to help identifying individuals who may benefit from weight self-stigma reduction interventions [44]. Indeed, it has been found that the assessment and consideration of weight self-stigma may help both patients and doctors to make treatment decisions and increase the beneficial effects of weight-reducing treatments [45]. Therefore, we believe that interventions that target weight self-stigma by cultivating a more accepting and compassionate way of dealing with those unwanted weight-related thoughts and emotions may improve people with obesity’s well-being [46].

### Future research

We think that further research on the applicability of the S-WSSQ in other Spanish-speaking countries is necessary. We believe that it would be interesting to examine the measurement invariance of the S-WSSQ among samples from different South American states (e.g., Argentina). Furthermore, future studies that investigate whether interventions focusing on weight self-stigma may enhance the health-related quality of life of patients with obesity are recommended. Finally, it is suggested to conduct invariance analyses via multigroup confirmatory factor analysis with the S-WSSQ to ensure the feasibility of comparisons between men and women.

### Limitations

The current study has at least four limitations. First, our sample size was not large enough (165 participants) to test sex invariance to make statements regarding differences between men and women. Consequently, there is a need to cross-validate the current results using an additional sample of individuals with obesity. Second, the ratio of men/women in our sample (65% women) was far different from what would be expected according to the prevalence of obesity in the Spanish population (22.8% for men, 20.5% for women, 1:1 ratio) [47]. Thirdly, our sample was only composed of patients with good adherence to a weight loss treatment which may have influenced the results of our study. Finally, the Weight Bias Internalization Scale (WBIS) was not used to test the convergent validity of the S-WSSQ [48]. The WBIS is a very useful instrument to measure the degree to which people apply weight-based stereotypes to themselves and base their self-evaluations on weight [49]. The WBIS has a Spanish version [50] and this scale has been validated to be used across different bodyweight categories [51], including obesity, although it was originally developed with normal-weight individuals [52]. Regardless, we believe that the S-WSSQ presented in this research may be used with guarantees for measuring weight self-stigma, as revealed throughout this article, although we shall advice professionals to be aware of the existence of other questionnaires.

## Conclusion

Despite these limitations, and based on the above-mentioned results, it is concluded that the S-WSSQ appears to be a useful instrument for measuring the extent to which individuals self-devaluate due to one’s identification with the group of people with obesity. We think that the psychometric evidence presented here may help researchers and clinicians in Spanish-speaking countries to have a reliable instrument that validly measures weight self-stigma.

### What is already known on this subject?

The Weight Self-Stigma Questionnaire (WSSQ) is an instrument specifically designed to measure weight self-stigma in overweight or obese populations. The WSSQ was originally developed in the United States and has been successfully validated in German, Chinese, Turkish, French, Italian, and Malaysian populations so far. The current study tries to extend this effort and presents the adaptation of the WSSQ in Spain.

### What does this study add?

This research is the first documented work, to the extent of our knowledge, that adapts the WSSQ to the Spanish population (S-WSSQ). According to the results reported in our research, the S-WSSQ has good psychometric properties and may be used by the Spanish-speaking scientific community for measuring weight self-stigma. We believe that cross-cultural studies, such as ours, are important in demonstrating the generalizability of questionnaires to measure weight self-stigma.

## Data Availability

Data will be provided upon request.
